# Better survival of patients with oligo- compared with polymetastatic cancers:  a systematic review and meta-analysis of 173 studies

**DOI:** 10.12688/f1000research.52546.4

**Published:** 2022-05-12

**Authors:** Fausto Petrelli, Antonio Ghidini, Michele Ghidini, Roberta Bukovec, Francesca Trevisan, Luca Turati, Alice Indini, Silvia Seghezzi, Veronica Lonati, Giovanna Moleri, Gianluca Tomasello, Alberto Zaniboni

**Affiliations:** 1 Oncology Unit, ASST Bergamo ovest, Treviglio (BG), Italy; 2 Oncology Unit, Casa di cura Igea, Milan, Italy; 3 Oncology Unit, Fondazione IRCCS Ca’ Granda, Ospedale Maggiore Policlinico, Milan, Italy; 4 Radiotherapy Unit, ASST Bergamo ovest, Treviglio (BG), Italy; 5 Surgery Unit, ASST Bergamo ovest, Treviglio (BG), Italy; 6 Nuclear Medicine Unit, ASST Bergamo ovest, Treviglio (BG), Italy; 7 Direzione socio sanitaria, Centro servizi, ASST Bergamo ovest, Treviglio (BG), Italy; 8 Oncology Unit, Fondazione Poliambulanza, Brescia, Italy

**Keywords:** cancer, oligometastases, survival, review, meta-analysis, tumours

## Abstract

**Background**: The modern concept of oligometastatic (OM) state has been initially developed to describe patients with a low burden of disease and with a potential for cure with local ablative treatments. We systematically assessed the risk of death and relapse of oligometastatic (OM) cancers compared to cancers with more diffuse metastatic spread, through a meta-analysis of published data.

**Methods**: PubMed, the Cochrane Library, and EMBASE were searched for studies reporting prognosis of patients with OM solid tumors. Risk of death and relapse were extracted and pooled to provide an adjusted hazard ratio with a 95% confidence interval (HR 95%CI).  The primary outcome of the study refers to overall mortality in OM vs. polymetastatic (PM) patients.

**Results**. Mortality and relapse associated with OM state in patients with cancer were evaluated among 104,234 participants (n=173 studies). Progression-free survival was better in patients with OM disease (hazard ratio [HR] = 0.62, 95% CI 0.57–0.68; P <.001; n=69 studies). Also, OM cancers were associated with a better overall survival (OS) (HR = 0.65, 95% CI 0.62-0.68; P<.01; n=161 studies). In colorectal (CRC), breast, non-small cell lung cancer (NSCLC) and renal cell carcinoma (RCC) the reduction in the risk of death for OM patients were 35, 38, 30 and 42%, respectively. Biliary tract and cervical cancer do not significantly better in OM stage likely for paucity of data.

**Conclusions**. Patients with OM cancers have a significantly better prognosis than those with more widespread stage IV tumors. In OM cancer patients a personalized approach should be pursued.

## Introduction

The vast majority of metastatic solid tumors are incurable, and despite the evolution of treatments, patients ultimately die because of their disease. The modern concept of oligometastatic (OM) state was initially developed in 1995 ^1^ to describe patients with a low burden of disease (e.g. 1 to 3-5 metastases) with a potential for cure with local ablative treatments. This assumption also relies on the hypothesis that metastatic spread follows a hierarchical pattern in time and number of localizations. ^2^ Large consensus on the definition and management of OM patients is currently lacking. Clinically, those cancers with a lower burden of metastatic disease have a favorable prognosis and they may be amenable of local treatment for the primary and distant tumors. Recently, infact, advances in imaging and local ablative therapies have permitted the treatment of these patients with additional locoregional treatment in addition to systemic therapies, and some patients may be cured and attain long term survival. ^3^ This scenario has been best elucidated in genitourinary, lung and melanomas. ^4, 5^ In these settings oligometastatic cancers may be treated in oligoprogressive sites continuing systemic therapy that control the remaining disease. One of the first published trials proving benefit of an aggressive local treatment of oligometastases was published in Lancet during 2019. In the SABR-COMET randomized study median overall survival (OS) was 28 months (95% CI 19-33) in the control group versus 41 months (26-not reached) in the stereotactic body radiotherapy to all metastases group (hazard ratio 0.57, 95% CI 0.30-1.10; P = .09). ^6^

The aim of this systematic review and meta-analysis was to investigate and establish the prognostic survival of OM compared to non-OM solid tumors. In particular, we evaluated if patients with oligometastatic solid tumors do better than patients with non-oligometastatic tumors.

## Methods

This study followed the Preferred Reporting Items for Systematic Reviews and Meta-analyses (PRISMA) guidelines.

### Search strategy and inclusion criteria

A comprehensive search was performed with the following terms: ( advanced or metastatic or recurrent or relapsed or synchronous or metachronous) and (site or oligo* or “oligometastastic” or oligorecurrence or oligoprogression or single or multiple or 1-3 or >3 or >4 or >5 or 1-2 or 1-3 or 1-5 or number) and (synchronous or metachronous or metastases or relapse or recurrence or progression) and (tumor or tumour or cancer or carcinoma or melanoma or sarcoma) and (“hazard ratio”) and (cox or multivariate or multivariable) and survival. We searched PubMed, the Cochrane Library and EMBASE for studies eligible for this meta-analysis published in English language from inception up to October 30 ^th^, 2020. To be eligible, studies needed to have evaluated survival of patients with OM cancers (1 up to 3/5 metastases regardless of anatomic sites) regardless of line of therapy and to provide data of outcome according to the number of OM sites used by each author. Studies were excluded if they enrolled less than 10 patients, pediatric subjects, and hematological diseases. Commonly we define polymetastatic cancer as any disease with more than three or more than five metastases. Studies were searched and screened independently by three authors (FP, MG and GT).

### Quality of studies and endpoints

The primary endpoint was overall survival (OS) and the secondary endpoint was progression-free survival (PFS). Quality assessment of the included studies was performed using the Newcastle-Ottawa Scale (NOS) for observational or retrospective studies ( http://www.ohri.ca/programs/clinical_epidemiology/oxford.asp). With NOS scale, studies were defined as poor, sufficient or good quality if scores (the sum of points attributed to each domain) were <6, 6 or 7-9 points, respectively.

### Data extraction and statistical analysis

The extracted data (from six reviewers) included the type of study, number of patients, cancer type, median age of included patients, performance status 0-1 (rate), treatment received, timing of oligometastases (synchronous or metachronous), number of OM sites used for comparison, and median follow up. Hazard ratios (HR) for OS and PFS with their 95% CIs, were extracted preferentially from multivariate analyses where available. The heterogeneity in the included studies was evaluated by the Chi-square-based Q-test and I ^2^ (I ^2^ = 0% to 25%, no heterogeneity; I ^2^ = 25% to 50%, moderate heterogeneity; I ^2^ = 50% to 75%, high heterogeneity; I ^2^ = 75% to 100%, extreme heterogeneity). When I ^2^ was larger than 50%, a random effects model was used; otherwise, the fixed effects model was used. Sensitivity analyses for OS were performed according to type of cancer, timing and number of oligometastases to find the potential heterogeneity among the included studies. If the number of studies was less than or equal to one, we did not carry out the subgroup analysis. The possibility of publication bias was explored by the Egger's and Begg's tests and Trim and Fill method. ^7, 8^ Begg's test explores bias with a funnel plot, conversely Egger's test is a linear regression of the effect estimates (OS) on their standard errors weighted by their inverse variance. The trim-and-fill method aims at estimating potentially missing studies due to publication bias in the funnel plot and adjusting the overall effect estimate. All analyses were performed using RevMan v.3 software. ^9^

## Results

**Figure 1.**
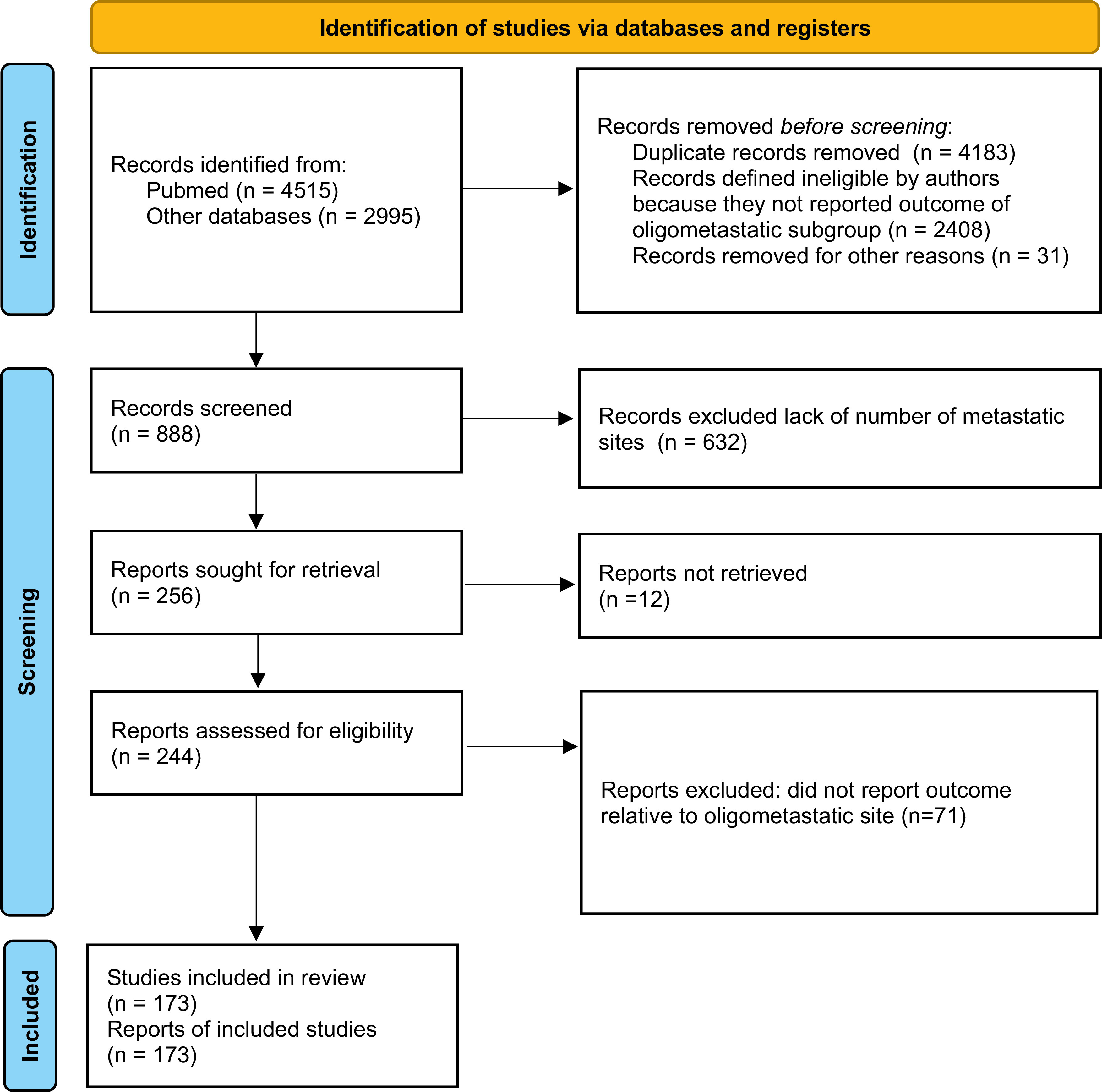
Preferred Reporting Items for Systematic Reviews and Meta-analyses (PRISMA) 2020 flow diagram showing the process of study inclusion.

Among the publications retrieved using electronic search (n = 7510), 173 studies were eligible for meta-analysis, for a total of 104,234 patients ^10^ ( Figure 1). Baseline characteristics of the included studies and treatments received are presented in Table 1.

**Table 1.** Characteristics of included studies.

Author/year	N° pts	Disease	Median age (years)	PS 0-1 (%)	Type of study	Median follow up (months)	Definition of OM (n° of lesions)/%	Site of OM	De novo / metachronous (%)	Treatment for OM (%)	OS (UVA or MVA)	PFS (UVA or MVA)	Quality
Morino/2020	232	Biliary	66	NR	Retrospective	12.6	1-3 (52)	Various	-	± Locoregional ± Systemic tx	UVA	-	5
Park/2017	134	Biliary	61	90	Retrospective	26	0-1 (90)	Various	-	CT (100)	MVA	MVA	6
Luzzago/2019	1592	Bladder	68	NR	Retrospective	NR	1 (44)	Various	-	CT ± S	MVA	-	5
Bates/2011	96	Breast	NR	NR	Retrospective	NR	1-2 (NR)	Various	-	CT (100)	MVA	MVA (TTP)	5
Blanchette/2018	154	Breast	56	NR	Retrospective	34	1 (55)	Various	25/75	CT (100)	MVA	-	7
Buhl/2018	140	Breast	62	NR	Retrospective	6.2	1 (40)	Various	-	CT (100)	-	MVA (TTP)	5
Co/2019	172	Breast	53	NR	Retrospective	NR	1-3 (96)	Various	100/0	Systemic tx ± S (100)	MVA	-	5
Gobbini/2018	16702	Breast	61	NR	Cohort	48.5	1-3 (92.6)	Various	28.5/71.5	-	MVA	-	8
Gu/2020	1888	Breast	NR	NR	Retrospective	NR	1 (NR)	Various	100/0	Various	MVA	-	5
Ivars Rubio/2019	263	Breast	59	81.4	Retrospective	44.9	1 (57.8)	Various	44.5/55.5	OT (19.8) CT (50.7) CT + Bio (29.5)	UVA	UVA	7
Kikawa/2019	134	Breast	63.5	85.8	Retrospective	NR	1-2 (73.9)	Various	24.6/75.4	Everolimus (100)	UVA	UVA	5
Kroger/2006	187	Breast	45.5	NR	Phase 3	63	1-2 (41.5)	Various	29.5/70.5	CT (100)	MVA	MVA	7
Le Scodan/2009	581	Breast	60.2	NR	Rerospective	39	1 (58.9)	Various	100/0	RT ± S	MVA	-	7
Leone/2017	9143	Breast	61	NR	Retrospective	13	1 (36.2)	Various	100/0	-	MVA	-	6
Lipton/2010	102	Breast	55.4	100	Retrospective	34	1-2 (NR)	Various	-	Trastuzumab (100) ± CT (88)	MVA	MVA (TTP)	6
Lobbezoo/2015	815	Breast	62.5	NR	Retrospective	37.1	1 (67)	Various	19/81	Systemic tx (100)	MVA	-	6
Neuman/2010	186	Breast	56	NR	Retrospective	52	1 (13)	Various	100/0	CT (100)	MVA	-	8
Nguyen/2012	692	Breast	60	68.9	Retrospective	22.8	1-4 (33.6)	Various	-	± Locoregional ± Systemic tx	MVA	-	6
Niikura/2012	314	Breast	51.9	90.4	Retrospective	33	1 (23.8)	Bone	100/0	Bisphosphonates	MVA	MVA	6
Park/2009	317	Breast	48	93	Retrospective	NR	1-2 (36)	Various	-	Various	MVA	-	5
Pons-Tostivint/2019	4276	Breast	60	NR	Retrospective	45.3	1-2 (77)	Various	100/0	Various	MVA	-	7
Ran/2020	49	Breast	50	NR	Retrospective	29	1-2 (NR)	Various	-	Trastuzumab based (100)	UVA	-	6
Rhu/2014	262	Breast	47	NR	Retrospective	29.6	1-2 (84.7)	Various	100/0	Various	MVA	-	6
Schneeweiss/2002	118	Breast	44	NR	Retrospective	48	1-2 (86)	Various	-	CT (100)	UVA	MVA	7
Wong/2019	483	Breast	49	NR	retrospective	66	1 (88)	Various	100/0	Systemic tx (100)	MVA	-	7
Smart/2019	66	BTC	76	55	Retrospective	21	1-2 (54)	Liver	-	RT (100)	MVA	MVA	6
Yin/2019	99	Cervix	53	51.6	Retrospective	11.6	1-3 (37.3)	Various	-	-	MVA	MVA	6
Afshari/2019	281	CRC	62	NR	Retrospective	NR	1 (85)	Various	-	-	MVA	-	5
Amikura/2017	342	CRC	NR	NR	Retrospective	52.7	1-4 (75.7)	Liver	63/37	S ± CT (100)	MVA	MVA	8
Aparicio/2016	282	CRC	80	100	Phase 3	69.8	1-2 (77.8)	Various	-	CT (100)	MVA	MVA	-
Araujo/2015	318	CRC	58	NR	Retrospective	60	1 (43)	Liver	-	S (100) + CT (37)	-	MVA	8
Bachet/2019	249	CRC	62.9	NR	Retrospective	28.7	1-3 (66)	Liver	79/21	S ± CT (100)	UVA	MVA (DFS)	6
Baldin/2021	221	CRC	62	NR	Retrospective	44.5	1-3 (75.6)	Liver	74.2/25.8	S ± perioperative tx (100)	MVA	MVA (TTR)	7
Beppu/2014	137	CRC	63	NR	Retrospective	NR	1-5 (NR)	Liver	-	CT ± S (100)	MVA	-	5
Blazer III/2008	305	CRC	57	NR	Retrospective	25	1 (32)	Liver	-	CT + S (100)	MVA	-	6
Brandi/2013	151	CRC	61.5	100	Retrospective	42	1 (61)	Lung / Liver	51/49	S ± CT (100)	-	MVA	8
Cardona/2013	1004	CRC	NR	NR	Retrospective	59	1 (42)	Liver	-	S (100)	MVA	-	8
Catalano/2009	255	CRC	67	92	Retrospective	45	1 (64)	Various	0/100	CT (100)	MVA	-	8
Chen/2010	255	CRC	NR	NR	Retrospective	11.9	1 (67)	Various	100/0	CT (67)	MVA	-	6
Comella/2005	254	CRC	NR	97	Pooled analysis of n=2 trials	NR	1 (55)	Various	54/46	CT (100)	MVA	MVA	5
Creasy 2018	907	CRC	64	NR	Retrospective	122	1 (52.7)	Liver	-	S + CT (100)	MVA	MVA	8
Cristobal/2014	250	CRC	69.5	81	Retrospective	NR	1-2 (90)	Various	65/35	NR	UVA	MVA	5
Daniel/2017	109	CRC	58.4	NR	Retrospective	NR	1-4 (46)	Liver	100/0	S ± CT (100)	MVA	-	5
de Geus-Oei/2006	152	CRC	61.5	NR	Prospective	17	1 (NR)	Liver	-	Various	UVA	UVA	6
Efficace/2008	742	CRC	62	92	Retrospective analysis	NR	1 (40)	Various	-	CT (100)	MVA	-	5
Faron/2015	810	CRC	63	83	Pooled analysis of n=4 trials	33	1-2 (85)	Various	100/0	CT ± S	MVA	MVA	6
Ghiringhelli/2014	409	CRC	65	59	Retrospective	32	1 (63)	Various	62/38	S ± CT (100)	MVA	-	6
Gu/2018	102	CRC	62	NR	Retrospective	NR	1 (36)	Liver	0/100	RFA ± CT (100)	MVA	-	5
Hebbar/2015	284	CRC	61.7	93	Phase 3	67	1 (48.9)	Various / Liver 83.5	67.7/32.3	S + CT (100)	-	MVA (DFS)	8
Hernandez/2016	522	CRC	64.5	NR	Retrospective	38.7	1 (65.7)	Lung	-	CT	MVA	MVA	6
Holliday/2017	34	CRC	56	NR	Retrospective	25	1-2 (100)	Various	100/0	SCRT	MVA	MVA	6
Huang/2020	179	CRC	62	NR	Retrospective	27.6	1 (51.5)	Lung	-	S (100)	MVA	-	6
Ishiguro/2006	111	CRC	NR	NR	Retrospective	43	1-3 (81)	Liver	100/0	S (100)	MVA	-	9
Kemeny/2014	169	CRC	55	NR	Retrospective	44.3	1-2 (47.3)	Various	66.8/33.2	S + HAI + Systemic tx (100)	-	MVA (RFS)	7
Konopke/2009	201	CRC	65	NR	Prospective	31	1-3 (94)	Liver	34.8/65.2	S (100)	MVA	MVA	6
Leal/2016	513	CRC	64.1	NR	Retrospective	37	1 (61.6)	Liver	100/0	S	MVA	MVA	7
Lin/2018	307	CRC	57.5	NR	Retrospective	31.7	1 (52.8)	Liver	66.4/33.6	S (100) ± RFA (10.1) ± Systemic tx	MVA	-	7
Liu/2010	52	CRC	70	NR	Retrospective	35.5	1 (58)	Liver	0/100	S + CT (100)	MVA	MVA (DFS)	6
Liu/2020	182	CRC	59.5	NR	Retrospective	32.5	1-3 (NR)	Liver	65/35	S ± CT	MVA	MVA (RFS)	6
Margonis/2015	334	CRC	50	NR	Retrospective	28.2	1-2 (NR)	Liver	54.8/45.2	S (100)	UVA	MVA (RFS)	6
Margonis/2017	389	CRC	58.4	NR	Retrospective	20.8	1-2 (NR)	Liver	57.3/42.7	S ± Ablation (18.5) ± CT (71.5)	-	MVA (DFS)	6
Margonis/2019	718	CRC	62.3	NR	Retrospective	30.4	1-3 (36.4)	Liver	51.2/48.8	S ± Systemic tx	MVA	-	6
Mise/2010	98	CRC	62	NR	Retrospective	60	1-3 (68)	Various	0/100	S (100)	MVA	-	8
Miyamoto/2015	78	CRC	65	92	Retrospective	19.2	2 (37)	Various	-	-	UVA	-	6
Narayan/2020	357	CRC	60	NR	Prospective	127	1 (NR)	Liver	100/0	S ± HAI	-	UVA (RFS)	9
Negri/2005	135	CRC	60.5	82.2	Case–control	76.8	1 (60.7)	Various	100/0	CT (100)	MVA	-	8
Neofytou/2015	140	CRC	NR	NR	Retrospective	33	1 (41.4)	Liver	71.4/28.6	± S ± Systemic tx	UVA	UVA	6
Nojiri/2011	31	CRC	63.3	NR	Retrospective	62	1-2 (64.5)	Lung	3.2/96.8	S (100)	MVA	-	8
Park/2016	221	CRC	62	NR	Prospective	34.7	1 (73.3)	Lung	13.1/86.9	S (100) ± CT (79.6)	UVA	MVA (DFS)	6
Parkin/2013	5853	CRC	64	NR	Retrospective	20	1-3 (79)	Liver	37/50	Surgery (100)	MVA	-	5
Peng/2017	150	CRC	58	NR	Retrospective	36	1 (NR)	Liver	67/33	S ± CT (100)	MVA	MVA (RFS)	6
Peng/2018	140	CRC	55	NR	Retrospective	13	1-3 (79)	Liver	70/30	MWA (100)	-	MVA	6
Prasanna/2020	513	CRC	63	NR	Retrospective	NR	1 (NR)	Various	51/49	S ± CT	UVA	-	5
Rhu/2017	410	CRC	60	NR	Retrospective	34	1 (63)	Liver	-	S (100)	MVA	-	6
Ruzzo/2012	59	CRC	NR	100	Retrospective	NR	1 (64)	Various	0/100	CT (100)	UVA	UVA	5
Sasaki/2016	485	CRC	58.5	NR	Retrospective	31	1-3 (65)	Liver	57/43	S / RFA (100)	MVA	-	6
Sasaki/2017	251	CRC	57	NR	Retrospective	30.3	1-3 (NR)	Liver	-	S ± CT (100)	MVA	-	6
Shimizu/2019	160	CRC	66	NR	Retrospective	64	1-3 (88)	Lung	18/83	S (100)	MVA	-	7
Sorbye/2012	342	CRC	NR	98.8	Subgroup analysis of prospective random	NR	1 (53)	Liver	34.5/64.5	Various	-	UVA	5
Souglakos/2009	168	CRC	59	NR	Retrospective	NR	1-2 (53)	Various	-	CT (100)	MVA	MVA	5
Stang/2016	113	CRC	70	NR	Retrospective	99	1-3 (77)	Liver	21/79	RFA (100) CT (95)	MVA	MVA	8
Stremitzer/2015	154	CRC	62	NR	Retrospective	34	1-2 (NR)	Liver	-	S (100)	MVA	MVA	6
Tarpgaard/2014	566	CRC	NR	96	Retrospective	37	0-1 (29)	Various	-	CT (100)	MVA	MVA	6
Van Cutsem/2004	1207	CRC	64	NR	Retrospective	NR	1 (25)	Various	76/24	CT (100)	MVA	-	5
Wang/2017	163	CRC	65	NR	Retrospective	37	1-2 (41)	Liver	82/18	S + CT (100)	MVA	MVA	6
Wei/2005	395	CRC	63	NR	Retrospective	31	1-3 (65)	Liver	51/49	S (100)	MVA	MVA	6
Xie/2018	332	CRC	58	NR	Retrospective	27.7	1 (65.2)	Various	72/18	Various	MVA	-	6
Yamashita/2017	74	CRC	59	NR	Retrospective	25	1 (74)	Liver	-	RFA/MWA + CT (100)	MVA	MVA	6
Zhao/2017	289	CRC	57	NR	Retrospective	34	1 (51)	Liver	66/34	S (100)	MVA	MVA	6
Ai/2017	3245	Esophageal	66	NR	Retrospective	NR	1-3 (NR)	Various	-	-	MVA	-	5
Hashimoto/2010	466	Gastric	60	85	Retrospective	NR	1-2 (NR)	Various	71.7/28.3	CT (100)	UVA	-	5
Kadokura/2013	208	Gastric	64	81.3	Retrospective	26.9	1 (69.7)	Various	-	CT	MVA	-	6
Kim/2008	304	Gastric	54	73.3	Retrospective	NR	1 (81.2)	Various	-	CT (100)	MVA	-	5
Kimura/2019	103	Gastric	67	NR	Retrospective	NR	1-2 (89)	Various	-	CT (100)	MVA	-	5
Kinoshita/2015	256	Gastric	64	NR	Retrospective	65	1-2 (82.8)	Liver	41.4/58.6	S (100) + CT (32.8)	MVA	UVA	8
Kondoh/2018	50	Gastric	67	72	Retrospective	NR	1-2 (74)	Various	-	CT (100)	UVA	-	5
Makiyama/2018	444	Gastric	75	NR	Retrospective	28.7	1 (37.3)	Various	-	CT (100)	-	MVA	5
Wang/2016	310	Gastric	58	100	Retrospective	NR	1 (70.6)	Various	-	Various	MVA	-	5
Wang/2018	321	Gastric	57	85	Retrospecive	32	0-1 (83)	Various	-	CT (100)	MVA	MVA	6
Liu/2015	981	HCC	52.5	NR	Prospective	32.7	1 (70.3)	Liver	-	± S (18.9) ± RFA (19.3) ± TACE (48.2)	-	MVA (RTDS)	7
Mazzaferro/2009	1556	HCC	55	NR	Retrospective	53	1 (26)	Liver	-	S (100)	MVA	-	7
Yoon/2010	52	HCC	49	NR	Retrospective	16.3	1 (75)	Lung	-	S (100)	MVA	-	6
Bollig/2020	283	Head & neck	59.8	NR	Retrospective	NR	1 (18.7)	Various	-	Various (100)	MVA	-	5
Lo/2017	120	Head & neck	NR	NR	Retrospective	51	1-3 (68.3)	LNs	-	S ± CT/RT	MVA	MVA (DFS)	8
Shen L/2015	505	Head & neck	NR	95	Retrospective	20	1 (18.8)	Various	100/0	CT ± RT (100)	MVA	-	6
Shen L/2015 (2)	312	Head & neck	46	89.1	Retrospective	16	1-3 (62.2)	Bone	43.9 / 56.1	Various	MVA	-	6
Shinoda/2020	48	Liposarcoma	43	NR	Retrospective	27.5	1 (52.1)	Various	-	Various	UVA (DSS)		5
Li/2019	100	Lung	60	96.1	Retrospective	39	1-3 (13.7)	Brain	100/0	TKI ± CT	UVA	-	7
Prelaj/2019	193	Lung	65	88	Retrospective	43	1-3 (NR)	Various	-	IT (100)	UVA	MVA	7
Bian/2016	401	Melanoma	NR	83	Retrospective	35	1-4 (87)	CNS	-	SBRT (100)	MVA	-	7
Iacono/2019	162	Melanoma	NR	82	Retrospective	48	1-2 (66)	Various	-	Systemic tx (100)	MVA	-	7
Lee/2009	2247	Melanoma	51	NR	Retrospective	22.5	1-2 (67.4)	Various	-	-	MVA	-	6
Moreau/2012	115	Melanoma	59	NR	Retrospective	19	1-3 (64)	LNs	93/7	S (100)	MVA	MVA (DMFS)	6
Seremet/2019	85	Melanoma	57	91	Retrospective	21	1-2 (44.7)	Various	-	ICIs (100)	MVA	UVA	6
Weide/2012	855	Melanoma	62	NR	Retrospective	25	1-2 (74.7)	Various	-	Various	MVA	-	6
Robelin/2019	162	Neuroendocrine	61	90	Retrospective	56	1-2 (85)	Various	49/51	Various	MVA	UVA	7
Jiang/2015	347	NPC	48	100	Retrospective	NR	1 (28)	Various	100/0	CT (57.9) CT + RT (68.8) RT (3.7)	MVA	-	5
Nie/2017	209	NPC	45	81.3	Retrospective	16.6	1 (49.8)	Various	24.9/75.1	CT (100)	UVA	UVA	6
Beau-Faller/2019	228	NSCLC	NR	42	Retrospective	NR	1-2 (65)	Various	0/100	TKI (100)	MVA	MVA	5
Ding/2017	85	NSCLC	66	75	Retrospective	9.8	1-3 (48)	Various	-	TKI (94)	MVA	MVA	6
Liu/2018	216	NSCLC	57	NR	Retrospective	7	1-3 (NR)	Brain	-	RT ± Systemic tx	MVA	-	6
Niibe/2016	61	NSCLC	NR	100	Retrospective	NR	1-2 (89)	SNC	18/82	SBRT or SRS (100)	MVA	-	5
Paccagnella/2006	324	NSCLC	62	93.7	Phase 2-3	19	1 (30.5)	Various	100/0	CT (100)	UVA	-	6
Park/2019	517	NSCLC	64	NR	Retrospective	NR	1 (57)*	Various	100/0	Various	MVA	-	5
Shin/2016	1024	NSCLC	64	85.5	Retrospective	42.2	1 (14.8)*	Various	-	Systemic tx (100)	MVA	-	7
Sperduto/2016	1481	NSCLC	NR	69.2	Retrospective	NR	1-4 (81)	Brain	-	Various	MVA	-	5
Takahashi/2019	41	NSCLC	67	82	Retrospective	19.6	1 (57)	Bone	100/0	Various (100)	UVA	UVA	6
Tambo/2020	95	NSCLC	72	77.9	Retrospective	8.8	1-2 (80)	NR	-	Pembrolizumab (100)	MVA	MVA	5
Liu/2020	125	Osteosarcoma	17	100	Retrospective	NR	1-2 (72)	Lung	-	CT ± S	-	MVA (PRS)	5
Bolm/2015	39	Pancreatic	NR	56	Retrospective	5	1 (56)	Various	-	RT (100)	MVA	-	6
Neron/2020	51	Phyllodes	56.4	95.9	Retrospective	62.1	1 (51)	Various	13.7/86.3	± S (31.3) ± RT (31.9) ± CT (72.5)	UVA	-	7
Armstrong/2007	686	Prostate	68.5	88	Retrospective	70	1-2 (88)	Various	-	CT (100)	MVA	-	9
Tablazon/2019	837	Prostate	76	NR	Retrospective	26	1 (NR)	Bone	-	-	MVA	-	7
Zhang/2020	160	Prostate	68	NR	Retrospective	47.2	1-4 (39.4)	Bone	-	RT + OT (100)	UVA	-	7
Alt/2011	887	RCC	62.5	85	Retrospective	33.6	2 (16.5)	Various	58/42	S (14)	MVA	-	8
Atzpodien/2003	425	RCC	NR	100	Retrospective	20	1-2 (82)	Various	0/100	Various	MVA	-	7
Beuselink/2014	200	RCC	59	85	Retrospective	67	1 (83)	Various	38/62	Systemic tx (100)	UVA	UVA	8
Bossé/2020	3454 1061	RCC	62 61	81 97	Retrospective 2-cohort	34 24.9	1 (19.5) 1 (17.2)	NR	-	TKI (100)	MVA	-	6
Cai/2017	143	RCC	60	NR	Retrospective	22	1 (72.7)	Various	-	TKI (100)	UVA	UVA	6
Dai/2020	146	RCC	56.5	71.9	Retrospective	36	1 (56.8)	Various	45.9/54.1	TKI (100)	MVA	MVA	6
Fay/2018	4736	RCC	59.2	100	Pooled analysis of n=12 phase 2-3 trials	NR	1 (NR)	NR	-	-	MVA	-	6
Fujiwara/2020	45	RCC	62	82	Retrospective	26.4	1 (36)	NR	-	Nivolumab (100)	UVA	-	6
Furubayashi/2017	59	RCC	67	85	Retrospective	NR	1-2 (86)	Various	-	TKI (100)	MVA	-	5
Gu/2017	184	RCC	54	NR	Retrospective	23.3	1 (85)	Various	-	Various	UVA	MVA	6
Ikeda/2018	116	RCC	66	NR	Retrospective	19.4	1 (66)	Various	-	TKI (100)	MVA	MVA	6
Ishihara/2017	118	RCC	NR	NR	Retrospective	NR	1 (NR)	Various	100/0	S	UVA	-	5
Keizman/2014	278	RCC	63	NR	Retrospective	55	1 (18)	Various	82/18	TKI ± S	UVA	UVA	8
Kim/2017	177	RCC	62	92.6	Retrospective	19.2	1-3 (NR)	Various	-	TKI (100)	MVA	UVA	6
Kwak/2007	186	RCC	58	86.5	Retrospective	17.4	1 (60.2)	Various	39.8/60.2	S ± ICIs	MVA	MVA	6
Kwak/2007 (2)	252	RCC	NR	61	Retrospective	17	1 (37)	Various	19/80	ICIs	MVA	MVA	6
Liou/2017	266	RCC	61	NR	Retrospective	12	1 (43)	Various	-	S (100)	MVA	-	6
Lu/2016	67	RCC	58	95.5	Retrospective	NR	1-4 (32.8)	Bone	-	TKI (100)	MVA	-	5
Richey/2011	188	RCC	60.8	65	Retrospective	6.9	1 (36)	Various	100/0	S + Systemic tx (100)	MVA	-	6
Schmidt/2005	321	RCC	51	NR	Retrospective	52	1-2 (60)	Various	-	Citokines (100)	UVA	-	7
Sharma/2015	93	RCC	61	76	Retrospective	13	1 (60)	Various	100/0	S ± Systemic tx (100)	MVA	-	6
Takagi/2019	71	RCC	66	99	Retrospective	NR	1 (45)	Various	-	TKI (100)	MVA	-	5
Thiery-Vuillemin/2017	224	RCC	67	82	Retrospective	18.3	1 (51)	Various	-	Systemic tx ± S (100)	UVA	-	6
Yamamoto/2018	51	RCC	65	80	Retrospective	NR	1 (45)	Various	-	TKI (100)	UVA	UVA	5
You/2016	325	RCC	NR	NR	Retrospective	NR	1 (37)	Various	55/45	S ± CT	MVA	MVA	5
Zhang/2019	287	RCC	56	NR	Retrospective	28	1 (53)	Various	-	S (100)	MVA	MVA	6
Dudek/2019	33	Sarcoma	55	NR	Retrospective	37	1-3 (72.7)	Lung	36/64	S (100)	UVA	-	7
Kawamoto/2020	98	Sarcoma	NR	NR	Retrospective	NR	1-2 (43.9)	Lung	-	Various	-	MVA (PMS)	
Nataraj/2016	102	Sarcoma	18	60	Retrospective	23	1-3 (31)	Lung	31/69	S ± CT (100)	MVA	MVA (EFS)	6
Shoushtari/2016	215	Sarcoma	56	26	Retrospective	175	1-2 (67)	Various	39/61	CT (100)	MVA	UVA	9
Stephens/2011	81	Sarcoma	43.5	NR	Retrospective	27	1-2 (33)	Lung	-	S (100)	MVA	-	7
Han/2011	61	SCLC	65	71	Phase 2	33.6	1-2 (NR)	Various	-	CT (100)	MVA	-	7
Shirasawa/2019	141	SCLC	70	62	Retrospective	NR	1-5 (34.7)	Various	100/0	CT (100)	MVA	-	5
Anraku/2003	133	Utherine	56	NR	Retrospective	40	1 (58)	Lung	6/94	S (100)	MVA	-	7
Bartosch/2016	130	Utherine	52	NR	Retrospective	48	1 (54)	Various	-	Various	MVA	-	7
Chen/2019	3981	Various	60.84	40.8	Retrospective	44.3	1 (16.5)	Various	-	Various (100)	MVA	-	7
de Baere/2015	566	Various	62.7	NR	Retrospective	35.5	1-2 (78)	Lung	-	RFA (100)	MVA	MVA	7
Dercle/2016	251	Various	52	NR	Retrospective	10.5	1-2 (40)	Various	-	ICIs (100)	MVA	-	6
Silva/2019	61	Various	66.3	NR	Retrospective	13.58	1-5 (35)	Spine	-	SBRT (100)	-	MVA (LC 1y)	6

*M1b single extratoracic organ; CNS, central nervous system; CRC, colorectal cancer; CT, chemotherapy; DMFS, distant metastasis–free survival; DSS, disease-specific survival; EFS, event-free survival; HAI, hepatic artery infusion; HCC, hepatocellular carcinoma; ICIs, immune checkpoint inhibitors; LNs, lymph nodes; MVA, multivariate analysis; MWA, microwave ablation; NPC, nasopharyngeal carcinoma; NSCLC, non-small-cell lung cancer; OM, oligometastatic disease; OS, overall survival; OT, ormonotherapy; PFS, progression-free survival; PMS, post-metastasis survival; PRS, postrelapse survival; PS, performance status; RCC, renal cell carcinoma; RFA radiofrequency ablation; RFS, relapse-free survival; RTDS, recurrence to death survival; S, surgery; RT, radiotherapy; SBRT, stereotactic body radiotherapy; SCLC, small-cell lung cancer; SRS, stereotactic radiosurgery; TACE, transarterial chemoembolization; TKI, tyrosine kinase inhibitor; TTP, time to progression; TTR, time to recurrence; tx, therapy; UVA, univariate analysis.

**Figure 2.**
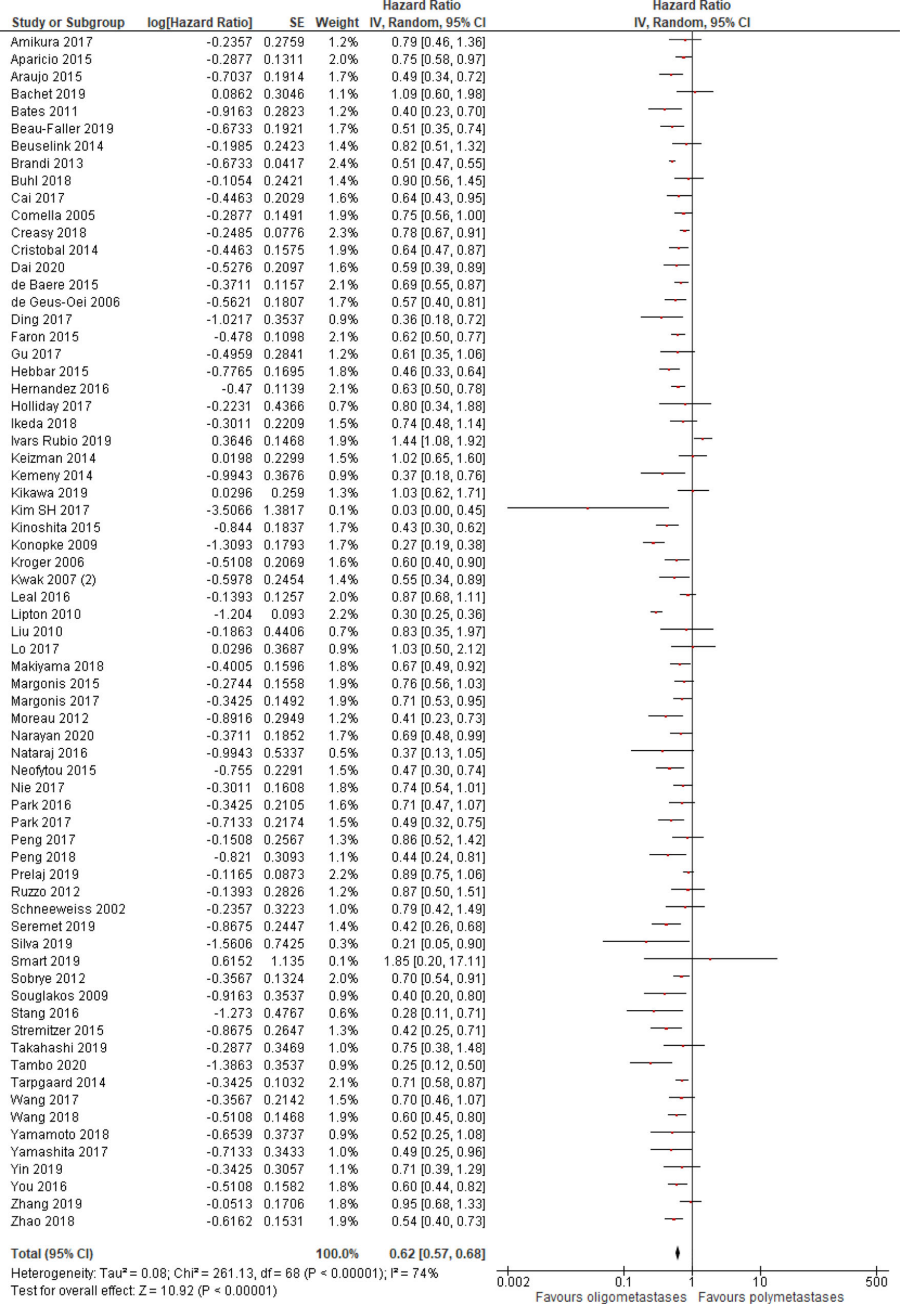
Progression-free survival of oligo- compared to non-oligometastatic cancers.

**Figure 3.**
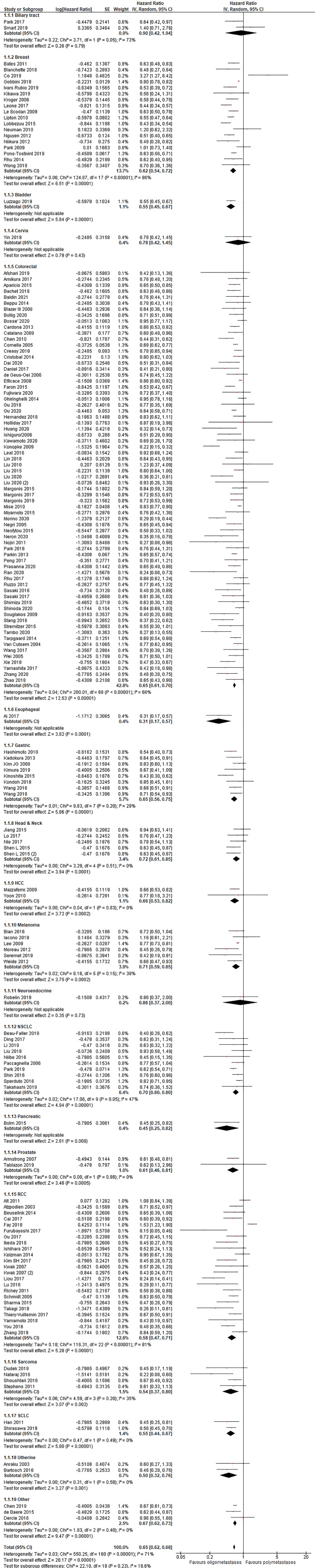
Overall survival of oligo- compared to non-oligometastatic cancers.

Progression-free survival was better in patients with OM disease (HR = 0.62, 95% CI 0.57–0.68; P < .01; n = 69 studies; Figure 2). Additionally, in the OS analysis, OM cancers were associated with a better OS (HR = 0.65, 95% CI 0.62–0.68; P < .01; n = 161 studies; Figure 3). Results were significant for all analyzed disease subgroups except biliary tract cancer and cervical cancer (only three studies included). In colorectal (CRC), breast, non-small cell lung cancer (NSCLC) and renal cell carcinoma (RCC), which constituted the more representative series, the reduction in the risk of death for OM patients were 35, 38, 30 and 42%, respectively ( Figure 3). Timing of onset (synchronous vs metacronous disease) did not influence the risk of death. Most studies reported OS analysis for up to three metastases (152 out of 161 studies). After exclusion of eight studies that reported outcomes for up to five metastases the final results remained unchanged (HR = 0.64, 95%CI 0.61-0.67; P < .01). No cut-off was associated with a better outcome (1 vs 2 vs 1-2 vs 1-3 metastases).

**Figure 4.**
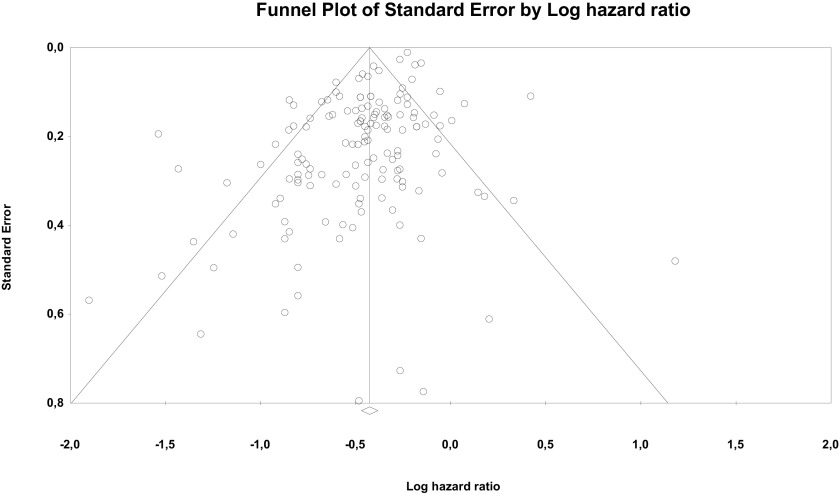
Funnel plot of publication bias for overall survival analysis showing standard error by log hazard ratio.

Risk of bias through Begg's funnel plot was not significant for the OS analysis. Conversely, Egger's test showed evidence of bias (P < .01) ( Figure 4). Trim and Fill analysis incorporated 29 missing studies. The overall effect measure (95% CI) based on this analysis was 0.7 (0.67-0.73), which became slightly weaker compared to the originally reported overall effect measure. Compared with cancers with more than three to five metastases, high-certainty evidence indicates OM tumors are associated with better prognosis in particular for CRC, breast, NSCLC and RCC. Despite the subgroup difference is not significant likely for less studies included in other groups, the results for these 4 cancers remain robust.

## Discussion

The definition of oligometastatic refers to malignancies with a limited metastatic spread which may be amenable of radical treatment for both primary and each distant site, and that generally have a better prognosis compared to polymetastatic cancers. A very recently published paper clearly explains the timely clonal evolution of somatic mutations and consequently the metastatic process of many cancer types. ^11^ It may be hypothesized that OM cancer is associated with a more indolent spread and therefore may represent a less fatal disease. With the expansion of the oncological armamentarium, many efforts have been made over the years to improve outcomes of patients with minimal metastatic burden. Advance in imaging may also have improved in the last years the diagnosis of oligometastases with the possibility of a more targeted approach toward primary tumor and every single oligometastatic site. This may have created a bias compared to older series, where less accurate imaging modalities were available and more frequent cases of oligometastases could now be overdiagnosed.

We have performed the most exhaustive systematic review of the literature to quantify the prognostic value of OM stage in various cancers. Overall, OM cancer patients have a risk of death and progression that is a third less than the polymetastatic counterpart. The OM state is frequently calculated as an independent favorable prognostic variable, which means that these patients do well independent from other clinical-pathological characteristics. The effect size was calculated from 173 studies including more than 100,000 patients. The final results were similar in all the most frequent histologies including CRC, breast cancer, NSCLC, RCC and sarcoma with inferior survival in OM gastric, melanoma and head and neck cancers.

Prognosis of OM cancer may be also different according to site of oligometastases. For example in CRC, breast and RCC lung metastases have a generally more favourable outcome than liver (or peritoneal ones in CRC). In our series, sites of oligometasteses were mixed or not described at all so a subgroup analysis was not performed. ^12^

There is also evidence from randomized clinical trials ^13- 15^ that ablative therapies improve survival in patients with OM cancer. For example, in some cancers small randomized studies ^13- 21^ already provide evidence of survival improvement in patients that received both systemic and local therapies compared to those that received systemic therapies alone. As a matter of fact, resection of colorectal cancer liver metastases nowadays represents an essential curative option and a primary endpoint in multiple clinical trails. ^13^ Furthermore, Gomez *et al.* ^14^ found that in OM NSCLCs, adding local consolidative therapy to active oligometastases and to primary disease may improve OS from 17 to 41 months. Also, in RCC the treatment of indolent lung metastases may permit delaying the start of systemic treatment and obtain an excellent control. ^15^ A large burden of evidence now supports local therapy for minimal oligoprogressive cancers treated with targeted therapies or immunotherapy. Here, metastases-directed therapy could delay the switch of systematic therapy by radical local treatment of all progressive metastatic sites. ^16, 17^ With the advent of immunotherapy, the combination of immune check point inhibitors and radiotherapy to single OM lesions may facilitate a potentiation of the immune response, increasing the chances of achieving an abscopal effect. This term describes an event in which focalized radiotherapy discharge systemic anti-tumoral action that can result in distant responses. ^18^ For example, in lung cancer the combination has a good safety profile and achieves high rates of local control and greater chances of obtaining abscopal responses than radiotherapy alone, with a relevant impact on outcome. ^19^ Oligometastatic cancers can also regarded as extended locoregional disease if, after proper conversion therapy, all sites of metastases and primary tumor may be radically resected with curative purposes. Such a strategy has been employed in largely incurable cancers as gastric and pancreatic carcinomas where selected cases with small liver-limited recurrences were managed with surgery. ^20, 21^ Melanoma and head and neck OM cancers are also associated with better prognosis. In these settings isolated recurrences (lymph nodes, lung nodules or brain metastases) may be radically treated with surgery or radiotherapy.

This meta-analysis has several limitations. First, our review does not evaluate the absolute benefit of any local treatment and the prognosis and management of oligoprogressive disease or down staged polymetastases to an OM state. Second, the literature search covered a large lifetime span and may include older series where radiological evaluation did not include more advanced modalities that can now increase the accuracy of oligometastases detection. Third, most of studies have an observational design and outcome was retrospectively analysed. Likely publication bias may influenced the prognosis of this population. Finally, the optimal number of lesions defining the OM state cannot be defined in this paper.

A consensus paper of EORTC and ESTRO societies attempted to provide definitions of various OM conditions either naïve or attained after therapy and either synchronous or metachronous. ^22^

Some large, randomized studies have included local therapies for OM cancers. An NRG Oncology randomized phase II/III trial study compares therapy with stereotactic radiosurgery and/or surgery with standard of care therapy alone in treating patients with breast cancer that has one or two locations in the body (limited metastatic) that are previously untreated. The PREST study will assess the efficacy of ablative radiotherapy (stereotactic body radiotherapy applied to all oligometastases) administered to all tumor sites (metastases and prostate if applicable), in oligometastatic hormone-sensitive prostate cancer patients. Finally, an ECOG-ACRIn phase III study compared standard chemotherapy to consolidative radiotherapy in patients with oligometastatic HER2 negative esophageal and gastric adenocarcinoma ( https://clinicaltrials.gov/ct2/show/NCT02364557; https://clinicaltrials.gov/ct2/show/NCT04115007; https://clinicaltrials.gov/ct2/show/NCT04248452). In all ongoing studies the aim is the optimal timing (after a good shrinkage during systemic therapy) and integration of systemic medical therapy and local ablation/resection with the scope of improving long-term survivals.

## Conclusions

In conclusion, this meta-analysis tried to quantify the prognosis associated with OM compared to cancers with more extensive diffusion. Based on our findings, we suggest that every metastatic patient should be accurately evaluated for the number of distant sites of disease, and a treatment strategy that involves both the primary and the metastases should be carefully considered. Patients could be reassured about their life expectancy and about the possibility of integrate both systemic and local therapy with the hope, in certain cases, for definitive cure. In others, focal treatment on the metastases may delay the immediate use of more toxic drugs (for example in elderly or indolent diseases). Also, we propose that these patients should be stratified when included in clinical trials and dedicated studies should be designed.

## Data availability

### Extended data

Mendeley Data: Extended data for ‘Better survival of patients with oligo- compared with polymetastatic cancers: a systematic review and meta-analysis of 173 studies’.

http://dx.doi.org/10.17632/8kycvdnp6v.1. ^10^

This project contains the following extended data:

- Supplementary Table 1: List of included studies.

### Reporting guidelines

Mendeley Data: PRISMA checklist for ‘Better survival of patients with oligo- compared with polymetastatic cancers: a systematic review and meta-analysis of 173 studies’.

http://dx.doi.org/10.17632/8kycvdnp6v.1. ^10^

Data are available under the terms of the Creative Commons Attribution 4.0 license (CC-BY 4.0).
