# Supplementary material for: Better survival of patients with oligo- compared with polymetastatic cancers:  a systematic review and meta-analysis of 173 studies
Source: F1000Res. 2022 May 12;10:423. Originally published 2021 May 27. [Version 4] doi: 10.12688/f1000research.52546.4 (PMC9106994; doi:10.12688/f1000research.52546.4)
Supplement: Peer review history

# Peer review: Reviewer response for version 4

**DOI:** 10.5256/f1000research.133866.r137591

**Published:** 2022-05-31

**Authors:** Dario Baratti

**Affiliations:** 1 Peritoneal Surface Malignancies Unit, Colorectal Surgical Division, Fondazione IRCCS Istituto Nazionale dei Tumori, Milan, Italy

The authors carefully revised their manuscript according to this reviewer's comments, resulting in a stronger paper.

Are the rationale for, and objectives of, the Systematic Review clearly stated?

Yes

Is the statistical analysis and its interpretation appropriate?

Yes

Are sufficient details of the methods and analysis provided to allow replication by others?

Yes

Are the conclusions drawn adequately supported by the results presented in the review?

Yes

Reviewer Expertise:

Peritoneal surface malignancies, advanced colorectal cancer

I confirm that I have read this submission and believe that I have an appropriate level of expertise to confirm that it is of an acceptable scientific standard.

# Peer review: Reviewer response for version 4

**DOI:** 10.5256/f1000research.133866.r137590

**Published:** 2022-05-13

**Authors:** Luca G. Campana

**Affiliations:** 1 Department of Surgery, Colorectal and Peritoneal Oncology Centre, The Christie NHS Foundation Trust, Manchester, UK; 2 Department of Surgery, Manchester University NHS Foundation Trust, Manchester, UK

The authors have satisfactorily addressed the majority of the requests, so I recommend the submission for indexing.

Are the rationale for, and objectives of, the Systematic Review clearly stated?

Partly

Is the statistical analysis and its interpretation appropriate?

Yes

Are sufficient details of the methods and analysis provided to allow replication by others?

Yes

Are the conclusions drawn adequately supported by the results presented in the review?

Partly

Reviewer Expertise:

General Surgery; Surgical Oncology; Clinical Research; Melanoma; Colorectal Cancer; Soft Tissue Sarcomas; Skin Cancer

I confirm that I have read this submission and believe that I have an appropriate level of expertise to confirm that it is of an acceptable scientific standard.

# Peer review: Reviewer response for version 3

**DOI:** 10.5256/f1000research.77989.r100190

**Published:** 2021-11-22

**Authors:** Luca G. Campana

**Affiliations:** 1 Department of Surgery, Colorectal and Peritoneal Oncology Centre, The Christie NHS Foundation Trust, Manchester, UK; 2 Department of Surgery, Manchester University NHS Foundation Trust, Manchester, UK

The authors of this report deserve praise for their extensive work. The data presented are intriguing and raise some intriguing questions.

- For the included studies, it would be interesting to know the comparator group and its tumour burden. This information would help to appreciate the magnitude of the differences in outcome.
- Additionally, one of the original questions raised with the introduction of the oligometastatic concept relates to the feasibility of local or locoregional treatment in a subset of patients with indolent disease. In this regard, I would present this information as a separate column in Table 1.
- Always in Table 1, the columns on OS and PFS present rather generic information. Therefore, I would suggest including the specific outcomes. Also, the column on Age and PS should be split. Finally, the content of the column “Type of study” should be homogenised.
- Further, the authors need to consider the time bias because modern imaging technologies increase the number of patients labelled as oligometastatic.
    
- It would be essential to distinguish between different types of oligometastatic disease (e.g., indolent progressive and minimal residual disease after previous treatments). In this regard, in Table 1, the column “De novo or metachronous” seems to provide this information, but it is not entirely clear.
- Please include the authors cited in Table 1 in the reference list.

**Minor comments**

Abstract

 Please revise and use terms consistently (e.g. avoid “overall mortality in OM”). In addition, the conclusions should be reformulated; in particular, the last sentence should be more focused on the results presented.

Introduction

 Please revise the language and, wherever possible, shorten the text (e.g. the first sentence is superfluous in this context). Also, please check some definitions such as “prognostic survival” and “with up to three to five metastatic sites.”  

Methods

 Please adjust the definition of polymetastatic accordingly.

Results

 Figure 1 *: *More than 2,000 reports were excluded from the analysis. The reason needs to be clarified.

 Table 1 should indicate more clearly the prevalence of patients with oligometastatic disease.

 Page 14: “Timing of onset did not influence the risk of death”. The authors should better explain this finding.

Discussion

 The discussion could be improved by discussing some general issues first (challenges in the definition of OM, changing scenario in terms of diagnostic tools and available treatments) and then presenting some reflections on the cancer types where the effect of OM on OS was more prominent. For instance, the criterium of OM disease has been long applied in surgical oncology for selecting patients with lung metastases for surgical resection or patients with peritoneal carcinomatosis for cytoreduction and intraperitoneal chemotherapy.

Are the rationale for, and objectives of, the Systematic Review clearly stated?

Partly

Is the statistical analysis and its interpretation appropriate?

Yes

Are sufficient details of the methods and analysis provided to allow replication by others?

Yes

Are the conclusions drawn adequately supported by the results presented in the review?

Partly

Reviewer Expertise:

Surgical oncology

I confirm that I have read this submission and believe that I have an appropriate level of expertise to confirm that it is of an acceptable scientific standard, however I have significant reservations, as outlined above.

# Response

**Published:** 2022-05-08

**Authors:** Fausto Petrelli

**Affiliations:** azienda ospedaliera Treviglio-Caravaggio, Italy

The authors of this report deserve praise for their extensive work. The data presented are intriguing and raise some intriguing questions.

- For the included studies, it would be interesting to know the comparator group and its tumour burden. This information would help to appreciate the magnitude of the differences in outcome.
**Data not available (comparator is the non-oligometastatic group but is not known site and number of metastases, for definition > 3-5 metastases).**
- Additionally, one of the original questions raised with the introduction of the oligometastatic concept relates to the feasibility of local or locoregional treatment in a subset of patients with indolent disease. In this regard, I would present this information as a separate column in Table 1.
**Data not available.**
- Always in Table 1, the columns on OS and PFS present rather generic information. Therefore, I would suggest including the specific outcomes. Also, the column on Age and PS should be split. Finally, the content of the column “Type of study” should be homogenised.
**OS and PFS are not generis but the exact outcomes (what is the meaning of specific outcomes?). Age and PS were split.**
- Further, the authors need to consider the time bias because modern imaging technologies increase the number of patients labelled as oligometastatic. **Sentence added in discussion.**
    
- It would be essential to distinguish between different types of oligometastatic disease (e.g., indolent progressive and minimal residual disease after previous treatments). In this regard, in Table 1, the column “De novo or metachronous” seems to provide this information, but it is not entirely clear.
**Data were not available. Only the information reported were extractable.**
- Please include the authors cited in Table 1 in the reference list.
**Due to the high number of studies, ref list is reported in a separated file.**

**Minor comments**

Abstract

 Please revise and use terms consistently (e.g. avoid “overall mortality in OM”). In addition, the conclusions should be reformulated; in particular, the last sentence should be more focused on the results presented.

**OK sentence modified.**

Introduction

 Please revise the language and, wherever possible, shorten the text (e.g. the first sentence is superfluous in this context). Also, please check some definitions such as “prognostic survival” and “with up to three to five metastatic sites.”  

**OK sentence modified. Sentences cancelled.**

Methods

 Please adjust the definition of polymetastatic accordingly.

**OK, sentence modified.**

Results

 Figure 1 *: *More than 2,000 reports were excluded from the analysis. The reason needs to be clarified.

**OK reason included.**

 Table 1 should indicate more clearly the prevalence of patients with oligometastatic disease. **Data already included in the table by the authors.**

 Page 14: “Timing of onset did not influence the risk of death”. The authors should better explain this finding.

**OK, sentence modified.**

Discussion

 The discussion could be improved by discussing some general issues first (challenges in the definition of OM, changing scenario in terms of diagnostic tools and available treatments) and then presenting some reflections on the cancer types where the effect of OM on OS was more prominent. For instance, the criterium of OM disease has been long applied in surgical oncology for selecting patients with lung metastases for surgical resection or patients with peritoneal carcinomatosis for cytoreduction and intraperitoneal chemotherapy.

**OK sentences added.**

# Peer review: Reviewer response for version 3

**DOI:** 10.5256/f1000research.77989.r100189

**Published:** 2021-11-22

**Authors:** Dario Baratti

**Affiliations:** 1 Peritoneal Surface Malignancies Unit, Colorectal Surgical Division, Fondazione IRCCS Istituto Nazionale dei Tumori, Milan, Italy

The authors have thoroughly addressed all my comments, resulting in a stronger paper.

Are the rationale for, and objectives of, the Systematic Review clearly stated?

Yes

Is the statistical analysis and its interpretation appropriate?

Yes

Are sufficient details of the methods and analysis provided to allow replication by others?

Yes

Are the conclusions drawn adequately supported by the results presented in the review?

Yes

Reviewer Expertise:

Peritoneal surface malignancies, advanced colorectal cancer

I confirm that I have read this submission and believe that I have an appropriate level of expertise to confirm that it is of an acceptable scientific standard.

# Peer review: Reviewer response for version 2

**DOI:** 10.5256/f1000research.58374.r93920

**Published:** 2021-09-22

**Authors:** Dario Baratti

**Affiliations:** 1 Peritoneal Surface Malignancies Unit, Colorectal Surgical Division, Fondazione IRCCS Istituto Nazionale dei Tumori, Milan, Italy

The authors present the results of their systematic review and meta-analysis assessing the prognostic impact of oligometastatic disease on adult patients with solid tumors, as compared with a more diffuse metastatic spread. Overall and progression-free survival were significantly longer in patients with 3-to-5 metastatic lesions, irrespective of anatomic site. This may sound quite obvious in modern oncology, but the authors were able to provide a large amount of clinical data to support such an assumption.

**Comments:**

**Abstract:**

- In the Introduction, the main topic of this literature review was described concisely but exhaustively.
- In the Conclusions, please use the term "oligometastatic disease (or OM)" instead of "oligometastases".

**Methods:**

- The methodology of literature search and data extraction, paper selection criteria, and statistical analyses are thoroughly described. The review was carried out according to international guidelines (PRISMA). Please, clarify if papers not in English language studies were included.
- Also, the Newcastle-Ottawa Scale (NOS) might be briefly described, as a number of readers may be not familiar with it.

**Results:**

- In the Results section, the authors state that the reduction in the risk of death for oligometastatic patients was 35%, 38%, 30%, and 42% for colorectal, breast, non-small cell lung cancer, and renal cell carcinoma (RCC), respectively. In another part of this section, they state that compared with cancers with more than three to five metastases, “high-certainty evidence indicates OM tumors are associated with better prognosis in particular for CRC, breast, NSCLC and RCC”. However, was such a difference significant? In agreement with Reviewer 1, I would suggest to group studies according to histology, and to graphically depict the risk of oligometastatic vs. more advanced disease for each of the four tumors mentioned above.
- Figure 1: Please, clarify in the Methods section what “Records marked as ineligible by automation tools” means.
- Figure 2 and 3: Please, refer to my comments about the Results section.

**Discussion:**

- The Discussion was improved according to the suggestions of Reviewer 1, resulting in a stronger manuscript. There is an additional concept that I would address in the paper: the fact that the site of metastatic disease may affect patient prognosis, in addition to the number of metastatic lesions. In colorectal cancer, peritoneal metastases are associated with worse prognosis as compared with liver metastases, and lung metastases are associated with better prognosis. Furthermore, specific areas within the same organ may be related to a worse prognosis, e.g. a metastasis involving the hepatic hilum may be worse than a subcapsular liver metastasis.

Are the rationale for, and objectives of, the Systematic Review clearly stated?

Yes

Is the statistical analysis and its interpretation appropriate?

Yes

Are sufficient details of the methods and analysis provided to allow replication by others?

Yes

Are the conclusions drawn adequately supported by the results presented in the review?

Yes

Reviewer Expertise:

Peritoneal surface malignancies, advanced colorectal cancer

I confirm that I have read this submission and believe that I have an appropriate level of expertise to confirm that it is of an acceptable scientific standard, however I have significant reservations, as outlined above.

# Response

**Published:** 2021-09-26

**Authors:** Fausto Petrelli

**Affiliations:** azienda ospedaliera Treviglio-Caravaggio, Italy

I have the comments of Reviewer 2:

- I changed the conclusion of the abstract as requested.
- I included a statement in the Methods section about the exclusion of non-English language papers and the NOS scale definition.
- I have modified Fig. 1.
- I have provided a new Fig. 5 with subgroup analysis according to disease histology.
- In the discussion section, I provided a brief discussion about the site of oligometastases (lung vs others), in particular for CRC.

# Response

**Published:** 2021-10-28

**Authors:** Fausto Petrelli

**Affiliations:** azienda ospedaliera Treviglio-Caravaggio, Italy

My responses to the comments of Reviewer 2:

- I changed the conclusion of the abstract as requested.
- I included a statement in the Methods section about the exclusion of non-English language papers and the NOS scale definition.
- I have modified Fig. 1.
- I have provided a new Fig. 5 with subgroup analysis according to disease histology.
- In the discussion section, I provided a brief discussion about the site of oligometastases (lung vs others), in particular for CRC.

# Peer review: Reviewer response for version 1

**DOI:** 10.5256/f1000research.55843.r87329

**Published:** 2021-06-30

**Authors:** Luca G. Campana

**Affiliations:** 1 Department of Surgery, Colorectal and Peritoneal Oncology Centre, The Christie NHS Foundation Trust, Manchester, UK; 2 Department of Surgery, Manchester University NHS Foundation Trust, Manchester, UK

The authors of this systematic review and meta-analysis assessed the influence of oligometastatic disease status on OS and PFS in adult patients with solid tumours. To do this, they carried out an extensive literature review including all types of studies with at least ten patients with any histology. Patients with OM disease were found to have significantly longer PFS and OS if they had CRC, BC, NSCLC, RCC, and sarcoma.

 The literature screening was conducted according to the standard recommendations and the subsequent analysis is methodologically robust. Going across several histotypes, the paper provides a big, and for certain aspects, unique picture of the prognosis of patients with OM. At the same time, however, it makes it challenging summarising and discussing the results.

 Here you can find some comments that you may find useful to improve this review:

- In the Abstract, I would mention the histotypes in which the OM status do not correlate with patient outcome. Also, in the Conclusions part, second sentence: this seems to be unrelated to the results presented and anyway not applicable in all cases (consider rephrasing/changing).
- The Introduction needs some input because sentences do not always follow a clear pattern. For instance, there are some general considerations regarding tumour progression, tumour staging according to the TNM, detailed results of a specific trial. It needs to be more homogeneous.
- Given the positive results with ablative therapies in patients with OM disease, the authors should explain what this meta-analysis adds to the literature.
- From the Introduction (and Methods) it is not clear what the definition adopted of OM disease is ("up to 3 to 5" metastatic sites). In this regard, is a patient with 6 liver metastases still considered "oligometastatic"?
- The great majority of the studies were retrospective in nature. This should be clearly stated and critically discussed as well.
- Did the authors detect any imbalance in treatment intensity between OM vs. non-OM patients?
- Table 1, 8th column: some of the included studies have "various" sites of OM. I think this information should be specified in order to be consistent with the inclusion criteria.
- The studies could be regrouped according to the histology. The same could apply to Figure 2 and Figure 3.
- The prognosis of patients with gastric cancer, melanoma, and head and neck cancer should be discussed in light of the results presented.
- In the Discussion, it is not entirely clear if the authors consider the OM status an opportunity to spare patients from systemic treatment or an opportunity to pursue combined treatment. Again, this should be discussed in light of the results presented.
- In the Discussion, the last paragraph seems more like a list of ongoing trials, including some form of local therapies over standard systemic treatment. How does this relate to the findings of the present study? Please discuss.

Are the rationale for, and objectives of, the Systematic Review clearly stated?

Partly

Is the statistical analysis and its interpretation appropriate?

Yes

Are sufficient details of the methods and analysis provided to allow replication by others?

Yes

Are the conclusions drawn adequately supported by the results presented in the review?

Partly

Reviewer Expertise:

Surgical oncology, locoregional therapies (limb perfusion/infusion, intraperitoneal chemotherapy, electrochemotherapy), melanoma, sarcoma, breast cancer, peritoneal malignancies, colorectal cancer.

I confirm that I have read this submission and believe that I have an appropriate level of expertise to confirm that it is of an acceptable scientific standard, however I have significant reservations, as outlined above.

# Response

**Published:** 2021-07-01

**Authors:** Fausto Petrelli

**Affiliations:** azienda ospedaliera Treviglio-Caravaggio, Italy

Reviewer 1: Luca Campana

 The authors of this systematic review and meta-analysis assessed the influence of oligometastatic disease status on OS and PFS in adult patients with solid tumours. To do this, they carried out an extensive literature review including all types of studies with at least ten patients with any histology. Patients with OM disease were found to have significantly longer PFS and OS if they had CRC, BC, NSCLC, RCC, and sarcoma.

 The literature screening was conducted according to the standard recommendations and the subsequent analysis is methodologically robust. Going across several histotypes, the paper provides a big, and for certain aspects, unique picture of the prognosis of patients with OM. At the same time, however, it makes it challenging summarising and discussing the results.

 Here you can find some comments that you may find useful to improve this review:

- In the Abstract, I would mention the histotypes in which the OM status do not correlate with patient outcome. Also, in the Conclusions part, second sentence: this seems to be unrelated to the results presented and anyway not applicable in all cases (consider rephrasing/changing).
  - **Author response: OK - requests accepted.**
- The Introduction needs some input because sentences do not always follow a clear pattern. For instance, there are some general considerations regarding tumour progression, tumour staging according to the TNM, detailed results of a specific trial. It needs to be more homogeneous.
  - **Author response: OK - sentences added or modified.**
- Given the positive results with ablative therapies in patients with OM disease, the authors should explain what this meta-analysis adds to the literature.
  - **Author response: Sentences added in 2nd paragraph of discussion.**
- From the Introduction (and Methods) it is not clear what the definition adopted of OM disease is ("up to 3 to 5" metastatic sites). In this regard, is a patient with 6 liver metastases still considered "oligometastatic"?
  - **Author response: Definition updated.**
- The great majority of the studies were retrospective in nature. This should be clearly stated and critically discussed as well.
  - **Author response: Considerations added in the limitations section.**
- Did the authors detect any imbalance in treatment intensity between OM vs. non-OM patients?
  - **Author response: This data was not reported.**
- Table 1, 8th column: some of the included studies have "various" sites of OM. I think this information should be specified in order to be consistent with the inclusion criteria.
  - **Author response: “Various” means that in those articles, sites of metastases were not specific. Only when explicitly reported they are included (e.g liver or lung). Specific comment in inclusion criteria added.**
- The studies could be regrouped according to the histology. The same could apply to Figure 2 and Figure 3.
  - **Author response: Table and Figure 2 (OS) arranged according to disease.**
- The prognosis of patients with gastric cancer, melanoma, and head and neck cancer should be discussed in light of the results presented.
  - **Author response: Sentences added in the Discussion.**
- In the Discussion, it is not entirely clear if the authors consider the OM status an opportunity to spare patients from systemic treatment or an opportunity to pursue combined treatment. Again, this should be discussed in light of the results presented.
  - **Author response: In the final paragraph, some sentences were added about this request.**
- In the Discussion, the last paragraph seems more like a list of ongoing trials, including some form of local therapies over standard systemic treatment. How does this relate to the findings of the present study? Please discuss.
  - **Author response: Discussion added.**

# Response

**Published:** 2021-07-02

**Authors:** Fausto Petrelli

**Affiliations:** azienda ospedaliera Treviglio-Caravaggio, Italy

- We have improved the Introduction and criteria for search.
- We have arranged in the Discussion section a specific discussion about particular settings of patients analysed and the main limitation of the paper (retrospective nature of studies).
- We also discussed the main meaning of the results: improved prognosis and treatment opportunities with locoregional therapies in an oligometastatic setting.
- Table was also ordered according to histology.
